# Paediatric Multiple Sclerosis: Update on Diagnostic Criteria, Imaging, Histopathology and Treatment Choices

**DOI:** 10.1007/s11910-016-0663-4

**Published:** 2016-06-06

**Authors:** I-Jun Chou, Huei-Shyong Wang, William P. Whitehouse, Cris S. Constantinescu

**Affiliations:** Division of Clinical Neuroscience, School of Medicine, University of Nottingham, Nottingham, UK; Division of Academic Child Health, School of Medicine, University of Nottingham, Nottingham, UK; Division of Paediatric Neurology, Chang Gung Children’s Hospital and Chang Gung Memorial Hospital, Chang Gung University College of Medicine, Taoyuan, Taiwan; Clinical Neurology Research Group, Division of Clinical Neuroscience, School of Medicine, University of Nottingham, Queen’s Medical Centre, Nottingham, NG7 2UH UK

**Keywords:** ADEM, CIS, Demyelination, MRI, NMOSD

## Abstract

Paediatric multiple sclerosis (MS) represents less than 5 % of the MS population, but patients with paediatric-onset disease reach permanent disability at a younger age than adult-onset patients. Accurate diagnosis at presentation and optimal long-term treatment are vital to mitigate ongoing neuroinflammation and irreversible neurodegeneration. However, it may be difficult to early differentiate paediatric MS from acute disseminated encephalomyelitis (ADEM) and neuromyelitis optica spectrum disorders (NMOSD), as they often have atypical presentation that differs from that of adult-onset MS. The purpose of this review is to summarize the updated views on diagnostic criteria, imaging, histopathology and treatment choices.

## Introduction

### What is Multiple Sclerosis?

Multiple sclerosis (MS) is an idiopathic inflammatory disorder characterized by demyelination and degeneration of the central nervous system (CNS) [1]. The pathogenesis is complex and not fully understood [2], but it is an autoimmune disease with contribution from genetic [3–5] and environmental factors such as infections, smoking and blood vitamin D levels [6–8].

MS is a chronic debilitating disease with disease onset typically in young adulthood, but in a minor proportion of patients, it starts in childhood. There are many functional domains of living, which can be impaired in a lengthy disease course, and a substantial proportion of adult MS patients are economically deprived as a result of unemployment [9].

### Clinical Course of Multiple Sclerosis

The heterogeneous clinical course of MS is broadly classified into three patterns: relapsing-remitting (RRMS), secondary progressive (SPMS) and primary progressive MS (PPMS). After the first attack suggestive of MS (clinically isolated syndrome (CIS)), more than 85 % of adult-onset patients experience a relapsing-remitting course; 10 % have a primary progressive onset with gradual worsening of function [10]. In contrast to a heterogeneous clinical presentation of clinical subtypes in adult MS, more than 98 % of paediatric-onset MS patients have a RRMS course [11–14]. In general, approximately two thirds of RRMS patients eventually evolve within two decades to SPMS [15•], which is characterized by a progressive worsening of disability with fewer relapses. The time to the transition to SPMS from the first symptom was shown to be associated with age of onset. Among RRMS patients whose first symptom was at age 20 years or younger, it was longer (25.8 years) than those with first symptom at 21 to 30 years (20.2) or at more than 30 years old (15.3) [16]. However, age at conversion to SPMS in patients with paediatric-onset course was 10 years younger than in those with adult-onset course (41 vs. 52 years old) [11].

### Impact of Multiple Sclerosis on Patients with Young-Onset Disease

A paediatric-onset course has been defined in various studies as having the first acquired CNS inflammatory demyelinating syndrome at age of less than 16 or 18 years. Some studies also include those with onset before 20 years. Paediatric-onset MS represents approximately 2 to 5 % of MS patients [11–13, 17, 18]. The acquired CNS inflammatory demyelinating syndromes comprise optic neuritis, transverse myelitis, monofocal or multifocal CIS; acute disseminated encephalomyelitis (ADEM); or neuromyelitis optica spectrum disorders (NMOSD). The incidence of acquired CNS inflammatory demyelination in the paediatric population is about 0.6 to 1.66 per 100,000 person-years [19–23]. About 32 to 50 % of children and adolescents with the first acquired demyelinating syndrome evolve to MS within 5 years [8, 20, 24–27]. The impact of MS on those affected young patients is enormous with multiple adverse medical and psychosocial outcomes. The common short-term medical problems include seizures, fatigue, depression, and impairment in mobility, hand function, and cognition. The proportion of children who need to repeat the grade in school after the first attack can be as high as 50 % in MS patients, especially those who had their disease onset after 11 years old [28]. The overall prognosis of childhood-onset MS tends to be worse than adult-onset MS. Generally, paediatric patients have a median time of 20 years to fixed disability and their median age at transition to SPMS is 10 years younger than in patients with adult-onset disease [11]. Of those with a disease onset before 20 years of age, there is a 1.55-fold risk of becoming bedbound from onset of progression compared to those with onset age larger than 30 years [15•].

### The Onset of Puberty Relevant to Paediatric Multiple Sclerosis

The exact causative factors for paediatric MS are unknown. There is no significant gender difference in the occurrence of MS before the age of puberty; however, female sex is a well-recognized risk factor afterwards [29]. The earlier onset of menarche was associated with a higher risk of MS in women in a Canadian population-based study [30]. In addition, for patients whose onset was at or before menarche, incidence of relapse was significantly higher during the peri-menarche period than the post-menarche period [31]. Furthermore, obesity was also associated with an increased risk for MS in female teenagers but not in boys [32].

### Difficulties in Diagnosing Multiple Sclerosis in Paediatric Populations

The clinical course of early-onset MS differs from that of adult-onset MS in many ways, and diagnosis is more challenging. Two diseases mimicking CIS or MS are ADEM and NMOSD. The differentiation of MS from ADEM and NMOSD is important for both treatment decision and prognosis prediction. Although longitudinally extensive myelitis is a key diagnostic feature of NMOSD, paediatric patients with MS and ADEM can also have a spinal cord lesion extending longer than three continuous vertebral segments. NMOSD can mimic MS or ADEM since many paediatric patients with NMOSD have large demarcated cerebral lesions [33, 34]. Up to about 30 % of adults and children with NMOSD have been reported to have oligoclonal bands (OCBs) in the cerebrospinal fluid (CSF) [35], which may complicate early clinical distinction of NMOSD from CIS.

Differentiating MS from MS mimics is of treatment and prognostic importance. Up to 30 % of ADEM patients eventually receive a diagnosis of MS after relapses and need a long-term disease-modifying treatment, while the others have a monophasic course. Exacerbation of the disease may occur if a patient with relapsing NMOSD is treated with some disease-modifying therapies for MS, such as fingolimod [36], natalizumab [37, 38] or interferon-β [39, 40].

**Fig. 1:**
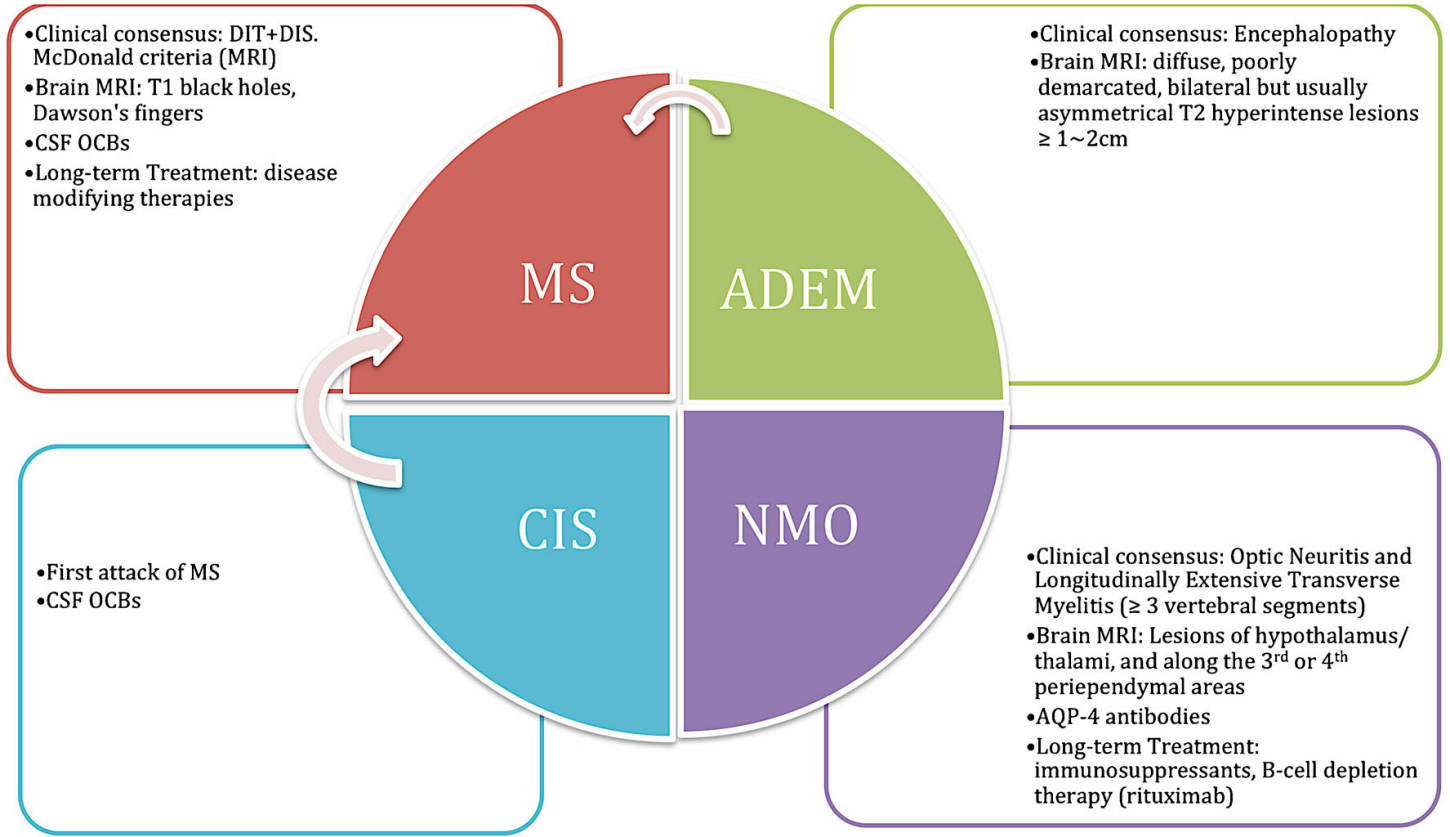
Key features to differentiate paediatric multiple sclerosis (*MS*), clinically isolated syndrome (*CIS*), acute disseminated encephalomyelitis (*ADEM*) and neuromyelitis optica spectrum disorders (*NMOSD*)

Figure 1 shows the features that distinguish between CIS, NMOSD, ADEM and MS.

### Diagnostic Consensus

An unambiguous early diagnosis is key to effective disease treatment. The updated diagnostic consensus for CIS, MS, ADEM and NMOSD led by International Pediatric Multiple Sclerosis Study Group [41•] is summarized in Table 1. There is no single clinical or paraclinical method than can ascertain a diagnosis of the aforementioned inflammatory demyelinating diseases, and there has to be no better explanation.

**Table 1:** Diagnostic consensus for multiple sclerosis (MS), acute disseminated encephalomyelitis (ADEM) and neuromyelitis optica spectrum disorders (NMOSD)

Classification	MS [42••]		ADEM [41•]	NMOSD [43••]	
	**1. ** CIS *Active* *Not active* **2.** RRMS *Active* *Not active* **3. ** SPMS *Active with progression* *Active without progression* *Not active with progression* *Not active without progression* **4. ** PPMS *Active with progression* *Active without progression* *Not active with progression* *Not active without progression (stable disease)*		**1. ** Monophasic **2. ** Multiphasic	**1. ** NMOSD with AQP4 **2. ** NMOSD without AQP4 or unknown AQP4-IgG Status	
	**CIS:** All are required [41•] • The first clinical presentation of a disease that shows characteristics of inflammatory demyelination that could be MS but has yet to fulfil criteria of dissemination in time [44] • Absence of a prior clinical history of CNS demyelinating disease (e.g. absence of past ON, TM, hemispheric or brain-stem syndromes) • No encephalopathy that cannot be explained by fever • The baseline MRI does not meet the diagnostic criteria for MS	**Definition of active and progression:** *Activity* is determined by • Clinical relapses in the absence of fever or infarction And/or • MRI activity (contrast-enhancing lesions; new or unequivocally enlarging T2 lesions assessed at least annually); • If assessments are not available, activity is “indeterminate” *Progression* is measured by clinical evaluation, assessed at least annually. If assessments are not available, activity and progression are indeterminate.	**Monophasic:** All are required [41•] • A first polyfocal clinical CNS event with presumed inflammatory demyelinating cause • Encephalopathy that cannot be explained by fever • No new clinical and MRI findings emerge ≥3 months after the onset • Brain MRI is abnormal during the acute phase (<3 month) • Typical brain MRI findings: • Diffuse, poorly demarcated, >1–2-cm lesions involving mainly the cerebral white matter • “Rare” T1 hypointense lesions in the white matter • Deep grey matter lesions can be present **Multiphasic** Two events consistent with ADEM attacks separated by ≥3 months	**NMOSD with AQP4** [43••] 1. At least one core clinical characteristic 2. Positive test for AQP4-IgG using best available detection method (cell-based assay strongly recommended) 3. Exclusion of alternative diagnoses	**NMOSD without AQP4 or unknown AQP4-IgG status** [43••] 1. At least two core clinical characteristics resulting from one or more clinical attacks and satisfying all of the following requirements: (a) At least one of the following: ON, acute myelitis with LETM or APS (b) Dissemination in space (>2 different core characteristics) (c) MRI requirements, if applicable (see below) 2. Negative test(s) for AQP4-IgG or testing unavailable 3. Exclusion of alternative diagnoses
	**RRMS:** [41•] Two clinical events, the first event can be CIS followed by another non-encephalopathic attack separated by more than 30 days or ADEM followed by a non-encephalopathic event separated by at least 3 months with clinical or MRI demonstrated dissemination in space. Age ≥12 years: 2010 McDonald MRI criteria [45] can be applied *Dissemination in space* Asymptomatic T2 lesion or gadolinium-enhanced lesions in each of two or more characteristic locations: • Periventricular • Juxtacortical • Infratentorium • Spinal cord *Dissemination is time* One of the following criteria (1) New T2 and/or gadolinium-enhancing lesion(s) on follow-up MRI, irrespective of the timing of the baseline scan (2) Simultaneous presence of asymptomatic gadolinium-enhancing and non-enhancing lesions at any time **PPMS** (very rare in paediatric population): [45] 1 year of disease progression (retrospectively or prospectively determined) plus 2 of 3 of the following (1) Dissemination is space in the brain - Presence of one or more T2 lesions in at least one area characteristic of MS (excluding the spinal cord) • Periventricular • Juxtacortical • Infratentorium (2) Dissemination in space in the spinal cord - Presence of two or more T2 lesions in the spinal cord (3) Presence of CSF OCBs and elevated IgG index			**Core clinical characteristics** Most common: 1. Optic neuritis (ON) 2. Acute myelitis (TM) 3. Area postrema syndrome (APS): episode of otherwise unexplained hiccups or nausea and vomiting Less common: 4. Acute brain stem syndrome 5. Symptomatic narcolepsy or acute diencephalic clinical syndrome with NMOSD-typical diencephalic MRI lesions 6. Symptomatic cerebral syndrome with NMOSD-typical brain lesions	**Supporting MRI** for NMOSD without AQP4 1. Acute optic neuritis: brain MRI normal or demonstrating only non-specific white matter lesions; OR optic nerve MRI with T2-hyperintense lesion or T1-weighted gadolinium-enhancing lesion extending over >1/2 optic nerve length or involving optic chiasm 2. Acute myelitis: spinal cord MRI showing attack-associated lesion extending >3 contiguous segments (LETM); OR >3 contiguous segments of focal cord atrophy in patients with prior history of acute myelitis 3. Area postrema syndrome: dorsal medulla/area postrema MRI lesion 4. Acute brain stem syndrome: peri-ependymal brain stem lesions

*AQP4* aquaporin-4, *APS* area postrema syndrome, *CIS* clinically isolated syndrome, *CNS* central nervous system, *CSF* cerebrospinal fluid, *IgG* immunoglobulin G, *LETM* longitudinally extensive transverse myelitis lesions, *MS* multiple sclerosis, *NMOSD* neuromyelitis optica spectrum disorders, *RRMS* relapsing-remitting multiple sclerosis, *OCBs* oligoclonal bands, *ON *optic neuritis, *PPMS* primary progressive multiple sclerosis, *SPMS* secondary progressive multiple sclerosis, *TM* transeverse myelitis

CIS, as defined in the adult MS population, refers to optic neuritis, brain stem syndrome, spinal cord syndrome or an isolated cerebral hemisphere syndrome [46, 47], and if the 2010 McDonald MRI criteria [45] are fulfilled with evidence of dissemination in time and in space, the diagnosis of MS can be made before the second clinical attack [46]. The 2010 McDonald MRI criteria [45] for MS can be applied in those onset at 12 years of age or older [41•]. The criteria were tested among patients with incident demyelination aged from 12 and up to 16 years [48] with a 76 % of positive predictive value [48], which is similar to the adult population (79 %)[46], while this value was lower (32 %) in those aged 11 years and younger [48].

### The Role of MRI

Before the advent of magnetic resonance imaging (MRI), the diagnosis of demyelinating diseases was primarily based on clinical presentation, physical examination, laboratory tests of both blood and CSF and electrophysiology. However, many other diseases, such as brain tumour, may manifest similarly to demyelinating lesions and therefore cause diagnostic uncertainty. MRI helps to exclude the other differential diagnoses without the need to resort to invasive procedures by providing topographic and pathological information about lesions. In addition, the evolution of the lesions, which can be clinically silent, becomes detectable. Furthermore, MRI can be used to monitor the suspected CNS areas before a lesion forms.

The role of MRI in the management of demyelinating diseases is becoming more and more important. MRI is an essential modality for the diagnosis of demyelinating diseases. It is also particularly useful in clinical trials. In addition, this technique provides a reliable prediction for disease outcome. Furthermore, there is a trend for increasing integration of MRI into MS treatment decisions [49].

The 2010 McDonald criteria suggest an MRI scan for the diagnosis of MS [45], which could also aid early diagnosis of MS for teenagers aged 12 years and older [48]. After the first attack (CIS or ADEM), patients having MRI evidence of new T2 lesions or gadolinium-enhancing lesions separated by a period of at least 30 days fulfil the MS diagnostic consensus. However, for those aged younger than 12 years, a minimum of two clinical events are mandatory to confirm the MS diagnosis. The first event can be CIS followed by another ADEM separated by more than 30 days or ADEM followed by a non-ADEM event separated by at least 3 months with a MRI demonstrating new lesions [41•]. It is important to note that NMOSD should be excluded, especially in an Asian population, when classifying patients using MRI criteria for MS.

Brain MRI is highly sensitive to white matter lesions. The MRI in MS (MAGNIMS) consensus recommended mandatory sequences for brain MRI includes (1) axial proton density and/or T2-fluid-attenuated inversion recovery (FLAIR)/T2-weighted, (2) sagittal two-dimensional (2D) or 3D T2-FLAIR and (3) 2D or 3D contrast-enhanced T1-weighted [50••]. Although axial diffusion-weighted imaging is optional, it potentially enables differentiation of an acute MS lesion (a lesion with gadolinium enhancement and increased diffusivity) from an acute ischaemic lesion (restricted diffusion) [51]. Spinal MRI is not recommended routinely in a patient without relevant spinal cord symptoms. However, it has value in increasing the sensitivity of MS diagnosis by detecting silent lesions [52] and also increases the specificity by excluding mimics [50••].

Contrast agent administration is commonly used to aid the diagnosis of MS, and it is most sensitive to active lesions characterized by blood-brain barrier breakdown. Active lesions are a common end point of treatment outcomes. There is some concern regarding patients who receive repeated gadolinium in serial MRI in a young population. A recent study revealed measurable gadolinium amounts in all autopsied brain samples, with higher concentration in the dentate nucleus and globus pallidus in five subjects exposed to two doses of gadolinium-based contrast agents compared to five unexposed subjects [53]. None of the examined adult subjects that received contrasts had a severely comprised renal function.

The MRI biomarkers to predict MS conversion in paediatric patients with any kind of CNS inflammatory demyelinating diseases are important. Verhey et al. prospectively analyzed 332 Canadian paediatric patients aged younger than 16 years old [54]. The presence of at least one “black hole” (a persistent hypointensity for more than 3 months on T1-wighted imaging) and at least one periventricular lesion (Dawson’s finger) was found to be sensitive to predict MS in paediatric patients with any kind of CNS inflammatory demyelinating diseases [54]. Presence of both parameters had a higher risk of MS conversion (hazard ratio [HR] 34.2) than presence of either one or more black holes (HR 20.6) or one or more Dawson’s fingers (HR 3.3) [54].

### Histopathology

A substantial body of literature describes the histopathology of white matter lesions in MS since the 1980s. Most studies described both paediatric-onset and adult-onset MS as showing histopathologic heterogeneity [55–59].

Although inflammatory demyelination is practically universal in the pathology of MS, four pathological patterns have been described [57]. Briefly, patterns I and II show typical confluent perivenous demyelinating plaques with T cell-mediated or T cell plus antibody-mediated processes, respectively. Patterns III and IV show features of oligodendrocyte dystrophy or extensive loss, respectively, in addition to lymphocytic infiltration [57]. These findings not only were nearly exclusively based on autopsy (more than 82 % lesions) and biopsy (18 % lesions) material from adult MS patients but also included a very small proportion of paediatric MS patients.

Despite the heterogeneity of lesions between patients, there seems to be a homogenous pattern in the same patient [57]. Metz et al. examined tissue sampled from different time points in 22 MS patients, and 21 of them showed the persistence of the original immunopathological patterns [59].

Comparing white matter lesions sampled for clinical diagnosis or autopsy between 19 paediatric and 12 adult MS [60••], an increased extent of acute axonal damage was noted in the paediatric group. The extent of acute axonal damage was positively correlated with a higher Expanded Disability Status Scale (EDSS; range, 0 to 10; higher scores indicate more severe disability and 10 indicates death) at the time of tissue sampling or autopsy. In addition, there were more lesions with a diameter more than 2 cm in early active demyelinating stages and more axonal damage in those with disease onset before 11 years old [60••].

Cortical lesions in MS have been described in adult patients but are rare in paediatric patients (66 vs 8 %) [61]. In adults, the cortical lesions are different from white matter lesions with a lack of inflammatory cell infiltration. The classification is based on the location of the lesions: subpial, intracortical and mixed white matter and grey matter (leukocortical) [62, 63].

### Oligoclonal Bands

The presence of intrathecal synthesis of antibodies (OCBs) is relevant to the diagnosis and prognosis in children with the first CNS inflammatory demyelinating disease. In a recent retrospective observational study of 357 children presenting with isolated optic neuritis, approximately 42 % had OCBs at disease presentation [64••]. Among these patients, 117/145 (81 %) patients who eventually converted to MS had positive CSF OCBs at presentation, whereas only 32/212 (15 %) patients who did not convert to MS had OCBs. In addition, the presence of OCBs in children might relate to patient age at onset. Studies showed that there was a higher frequency of CSF OCBs among paediatric MS with disease onset at 11 years and older (68 %) than those with a younger-onset age (43 %; odds ratio 2.6 with a 95 % confidence interval 0.8 and 8.8) [65]. Although the presence of OCBs was less frequent in MS patients diagnosed before puberty, after serial lumbar punctures, many of these patients eventually had positive OCBs in CSF [60••, 65, 66]. The presence of OCBs in children with the first CNS inflammatory demyelination is a predictor for a later relapse and MS diagnosis. However, the presence OCBs in CSF did not seem to be a good predictor in Asian paediatric patients [24, 67].

The positive rate of OCBs at the first attack varies in different paediatric population ranging from 44 to 83 % among those converted to MS [24, 64••, 67–69]. The difference is probably due to the timing of lumbar puncture in respect to disease onset, different laboratory techniques and the genetic background. The large cohort of genetic studies usually included paediatric-onset patients for disease course analyses. A recent large multinational genome-wide association study (GWAS) in 6950 adult MS patients (multiple countries of UK, Europe, USA and Australia; age range 2 to 72 years old) confirmed that genetic factors underlie the positivity of OCBs in CSF with the major histocompatibility complex and immunoglobulin heavy chain region being the most important area of interest. Thus, it appears that the frequency and diagnostic value of OCBs show no major differences between the paediatric- and adult-onset MS populations.

### Serological Tests

Diagnostic uncertainty often exists in paediatric patients. For instance, paediatric MS patients can have longitudinally extensive transverse myelitis, which alone does not exclude MS [70••]. Serological biomarkers are clinically useful for differentiating difficult cases and for disease course prediction. On the one hand, a negative result for autoantibodies against aquaporin-4 (AQP4) increases the confidence of MS diagnosis [71, 72]. On the other hand, testing for autoantibodies against myelin oligodendrocyte glycoprotein (MOG) potentially facilitates decision-making for long-term treatment [73, 74].

#### Autoantibodies to Aquaporin-4

Autoantibodies against AQP4 are specific for NMOSD, and a positive result of these antibodies in the serum or CSF is helpful to confirm NMOSD [75]. A growing body of literature confirmed the high specificity of autoantibodies to AQP4 for NMOSD diagnosis (85–100 %), although the sensitivity of various assays is moderate (33–91 %) [71, 72]. However, unavailability of immunoassays for AQP4 in many clinical settings hinders its timely usage in urgent clinical practices. The clinicians should be also aware of the false-positive results using enzyme-linked immunosorbent assay (ELISA) for sera and a need for confirmation with live cell-based assay [72, 76].

#### Autoantibodies to Myelin Oligodendrocyte Glycoprotein

The presence of serum antibodies to MOG was shown to be more frequent in patients with a non-MS course, especially in adults [77, 78]. Approximately a quarter of paediatric patients with MS and ADEM are positive for anti-MOG antibodies, which is relatively rare in adult MS [73, 79]. The titres of autoantibodies to MOG in paediatric MS patients are usually low and transient if serial serology tests were performed [80]. Less than half of AQP4-seronegative NMOSD patients have positive serum anti-MOG antibodies [81]. NMOSD patients with positive anti-MOG antibodies tend to involve younger adults with a usually monophasic, steroid-dependent clinical course [43••, 82]. MOG autoantibodies are not disease-specific since they can also be observed in a proportion of patients with epilepsy and also in healthy controls [43••]. However, the presence of MOG antibodies in the sera can potentially guide treatment in patients with demyelinating disease. For instance, anti-MOG-seropositive optic neuritis has been noticed to be steroid-sensitive and also steroid-dependent [83, 84].

Recent research suggests that the presence of antibodies to MOG in paediatric patients with the first acute CNS inflammatory demyelination is less likely to be MS and may need longer duration of steroid maintenance treatment after pulse therapy to prevent early relapses. However, there is no standardized or commercialized assay available and antibodies to MOG have not been tested in large populations of paediatric patients.

### Cognition in Paediatric Multiple Sclerosis

Most paediatric MS patients experience a recovery of functional deficit, such as problems with gait, vision, bladder and bowel function, within 12 months from the first attack [70••]. Nevertheless, the cognitive impairment may remain. Cognitive impairment was shown to affect 31 % of paediatric patients and the school activities and achievements in 28 %, within 2 years after disease onset [84]. At 5-year follow-up, half of patients showed cognitive deterioration, and cognitive deficit in visual-spatial learning and in expressive language was observed in 38 % of 48 patients studied [85].

### Treatment: Implications and Options

The treatment scheme of paediatric MS includes the acute management and long-term prevention for relapses. Although there has been a lack of randomized controlled trials in paediatric patients, many immunomodulatory and immunosuppressive agents have been used [85]. Corticosteroids are the most commonly used agents for acute disabling relapses; the most commonly used is intravenous methylprednisolone (30 mg/kg/day) for 3 to 5 days with or without oral steroid tapering according to clinical disability persistence. For persistent life-threatening conditions such as respiratory compromise, plasma exchange (5 to 7 cycles in 2 weeks) has been shown to prevent fatal outcomes and decreased the disability severity [86]. Alternative managements including intravenous immunoglobulin G (IVIG) [87] or cyclophosphamide [88] have been reported.

Immunomodulators are classified into first and second line with different regulatory approvals between countries, although there is no evidence to support the optimal order [89]. The first-line disease-modifying therapies (DMTs) for paediatric MS include interferon (IFN)-1 beta and glatiramer acetate (GA) [87, 88, 90, 91]. Whereas IFN was more commonly prescribed than GA, both agents result in a 24–40 % reduction in disability progression in paediatric and adult RRMS and their long-term side effects were similar between paediatric and adult MS [87]. The clinical efficacy is not different between IFN and GA, but IFN reduces the MRI lesion burden more than GA [92].

The long-term safety of the first-line DMTs is good, as no secondary malignancy or progressive multifocal leukoencephalopathy (PML) has been reported in paediatric or adult users. The adverse effects to interferon-beta 1b in paediatric MS patients were similar to adults, with flu-like symptoms in one third of patients, abnormal liver function profile in one fourth and injection site reaction in one fifth [87, 91]. Similar adverse events were reported in about 30 % of GA paediatric users [87]. However, poor tolerability of first-line DMTs was reported to occur in about one in eight paediatric users [90]. Approximately 30 % of all paediatric patients treated with first-line DMTs were non-responders, with more than one clinical relapse per year or MRI changes while on therapy [90].

It is usually reasonable to switch to a second-line DMT when there is breakthrough disease activity, such as disease relapse or new MRI T2 lesions. The common choices for paediatric patients include intravenous natalizumab and oral fingolimod.

#### Natalizumab

Natalizumab is a humanized recombinant monoclonal antibody against the α4-integrin, which diminishes leukocyte migration across the blood-brain barrier from the peripheral blood into the CNS [93]. Natalizumab was shown to reduce annual relapse rate by 68 %, reduced cerebral MRI disease activity and sustained EDSS progression at 2 years among adult RRMS patients [94, 95]. It has also been shown to be potentially effective in paediatric patients. Kornek et al. reported 20 paediatric RRMS patients who had reduced annual relapse rates and new T2 lesions after natalizumab administration [96]. However, they also found relapse activity within 6 months in six of eight patients after discontinuation of natalizumab therapy [96]. Ghezzi et al. also confirmed the efficacy in registered Italian paediatric patients receiving natalizumab and showed that no evidence of disease activity (NEDA) occurred in 58 % of cases compared to the pre-treatment status [97] and side effects were mild and tolerable [98].

The risk of progressive multifocal leukoencephalopathy (PML) has been studied in adult RRMS patients, and the risk factors include prolonged use of natalizumab (>2 years), prior immunosuppressive treatment and the presence of antibodies to the John Cunningham virus (JCV) [99, 100]. The seroprevalence of JCV in German children and adolescents with MS or other neurological conditions was shown to be around 30 %, which was about half of the seropositivity in adults [101, 102]. A higher anti-JCV antibody titre may predict higher risk of PML [99], and therefore, serial follow-up of viral titres or watching for seroconversion from seronegative subjects can preclude the high-risk patients. In addition, Huppke et al. studied the prevalence of anti-JCV antibodies in German paediatric MS patients and found at least twice as high as that reported in studies of non-MS children [103]. The risk of malignancy is not very high among adult natalizumab users (about <0.1 %, 7/24 cases with malignancies were breast cancer) [104] whereas careful surveillance is warranted in paediatric users who were in transition to young adulthood.

#### Fingolimod

Fingolimod is the first approved oral DMT for MS patients, which has been shown to be moderately effective [105]. Its mechanism is to prevent the egress of lymphocytes from lymph nodes via binding to the receptor for sphingosine-1-phosphate (S1P) on lymphocytes [106]. The short-term side effects in 17 Brazilian paediatric RRMS patients were similar to those in adult patients: asymptomatic bradycardia, genital herpes and isolated infection of the upper respiratory tract or urinary tract [107, 108]. Although the long-term experience in paediatric MS is still limited, rebound inflammatory effects on cerebral lesions in a 19-year-old childhood-onset patient indicate the need of a cautious withdrawal at a slower pace, if discontinuation of fingolimod is necessary [109].

#### Newer Oral Disease-Modifying Therapies

Teriflunomide was the second oral disease-modifying agent approved for adult MS [110]. Its primary mechanism is hypothesized to relate to inhibition of the proliferation of stimulated T or B lymphocytes [111]. Dimethyl fumarate (DMF, also called BG-12) is the latest oral disease-modifying agent in adults since 2013 [112]. The mechanism by which DMF exerts its therapeutic effect in MS is unknown, but it may affect the metabolism and signalling of immune cells. The safety and efficacy of these two agents have not yet been evaluated in paediatric MS patients.

## Conclusions

The formal diagnosis of MS should always take into consideration the clinical, imaging and serology or, if necessary, histopathology findings. In addition, no better explanation for the neurological syndrome should be present. When there is diagnostic uncertainty, serum antibodies against aquaporin-4 or MOG may be helpful in diagnosis (by ruling out the alternative diagnosis of NMOSD) and treatment. Optimal long-term DMT treatment in paediatric MS has not been well established, but there is experience of first-line treatment with IFN and GA and these are safe. There is also emerging experience with second-line DMT such as natalizumab and fingolimod; however, close monitoring for severe adverse events including PML and malignancy is warranted.
